# Supplementary material for: MetaQTL: a package of new computational methods for the meta-analysis of QTL mapping experiments
Source: BMC Bioinformatics. 2007 Feb 8;8:49. doi: 10.1186/1471-2105-8-49 (PMC1808479; doi:10.1186/1471-2105-8-49)
Supplement: Additional File 7 — MetaQTL Package : jar file and tutorial. This Zip archive contains both the MetaQTL JAR file and the files of the tutorial. [file 1471-2105-8-49-S7.zip › org.inra.metaqtl/doc/org/metaqtl/bio/entity/Locus.html]

Locus


|  |  |  |
| --- | --- | --- |
| **Overview** \| **Package** \| **Class** \| **Use** \| **Tree** \| **Deprecated** \| **Index** \| **Help** | |  |
| **PREV CLASS**   **NEXT CLASS** | **FRAMES**    **NO FRAMES**     **All Classes** |  |
| SUMMARY: NESTED \| FIELD \| CONSTR \| METHOD | DETAIL: FIELD \| CONSTR \| METHOD |  |


---


## org.metaqtl.bio.entity Class Locus

```
java.lang.Object
  org.metaqtl.bio.entity.BioEntity
      org.metaqtl.bio.entity.BioEntityContainer
          org.metaqtl.bio.entity.AlleleContainer
              org.metaqtl.bio.entity.Locus
```

**All Implemented Interfaces:**: IBioAdaptable, IBioEntity, IBioLocus

**Direct Known Subclasses:**: Marker, QTL

---

``` public abstract class Locus extends AlleleContainer implements IBioLocus ```

---

| **Field Summary** | |
| --- | --- |
| `protected  ILGroupPosition` | `position` |

**Fields inherited from class org.metaqtl.bio.entity.BioEntityContainer**

`entities`

**Fields inherited from class org.metaqtl.bio.entity.BioEntity**

`name, parent, properties`

**Fields inherited from interface org.metaqtl.bio.IBioLocus**

`MARKER, QTL, TypesString`


**Constructor Summary**

`Locus()`

`Locus(java.lang.String name,
IBioEntity parent)`


| **Method Summary** | |
| --- | --- |
| `IBioLGroup` | `getGroup()` |
| `static IBioAdapter` | `getLocusAdapter()` |
| `ILGroupPosition` | `getPosition()` |
| `static Locus` | `newLocus(int locusType)`             Returns a new Locus from the given locus type. |
| `static Locus` | `newLocus(java.lang.String string)`             Returns a new Locus from the given locus type. |
| `void` | `setGroup(IBioLGroup group)` |
| `void` | `setPosition(double position)`             Set the position of the locus on the linkage group in static mode i.e without uncertainty. |
| `void` | `setPosition(double position, double sd)`             Set the position of the locus on the linkage group in estimate mode i.e with uncertainty. |
| `void` | `setPosition(ILGroupPosition position)` |

**Methods inherited from class org.metaqtl.bio.entity.AlleleContainer**

`addAllele, alleles, getAllele, getAlleleNumber, getType, removeAllele`

**Methods inherited from class org.metaqtl.bio.entity.BioEntityContainer**

`addEntity, entities, entityNumber, getEntity, removeEntity`

**Methods inherited from class org.metaqtl.bio.entity.BioEntity**

`getName, getParent, getProperties, newBioEntity, setName, setProperties`

**Methods inherited from class java.lang.Object**

`clone, equals, finalize, getClass, hashCode, notify, notifyAll, toString, wait, wait, wait`

**Methods inherited from interface org.metaqtl.bio.IBioLocus**

`addAllele, alleles, getAllele, getAlleleNumber, getLocusType, getType, removeAllele`

**Methods inherited from interface org.metaqtl.bio.IBioEntity**

`getName, getParent, getProperties, setName, setProperties`

**Methods inherited from interface org.metaqtl.bio.IBioAdaptable**

`getBioAdapter`

**Field Detail**

### position

```
protected ILGroupPosition position
```


**Constructor Detail**

### Locus

```
public Locus()
```

---


### Locus

```
public Locus(java.lang.String name,
             IBioEntity parent)
```

**Parameters:**: `name` -: `parent` -


**Method Detail**

### newLocus

```
public static Locus newLocus(int locusType)
```

:   Returns a new Locus
    from the given locus type.

    :   **Parameters:**: `locusType` - **Returns:**

---


### newLocus

```
public static Locus newLocus(java.lang.String string)
```

:   Returns a new Locus
    from the given locus type.

    :   **Parameters:**: `string` - **Returns:**

---


### getLocusAdapter

```
public static IBioAdapter getLocusAdapter()
```

:   **Returns:**

---


### getGroup

```
public IBioLGroup getGroup()
```

:   **Specified by:**: `getGroup` in interface `IBioLocus` **Overrides:**: `getGroup` in class `AlleleContainer`

---


### setGroup

```
public void setGroup(IBioLGroup group)
```

:   **Specified by:**: `setGroup` in interface `IBioLocus` **Overrides:**: `setGroup` in class `AlleleContainer`

---


### getPosition

```
public ILGroupPosition getPosition()
```

:   **Specified by:**: `getPosition` in interface `IBioLocus` **Specified by:**: `getPosition` in class `AlleleContainer`

---


### setPosition

```
public void setPosition(ILGroupPosition position)
```

:   **Specified by:**: `setPosition` in interface `IBioLocus` **Specified by:**: `setPosition` in class `AlleleContainer`

---


### setPosition

```
public void setPosition(double position)
```

:   Set the position of the locus on the linkage group in
    static mode i.e without uncertainty.

    :   **Specified by:**: `setPosition` in interface `IBioLocus`
    :   **Parameters:**: `position` - the locus position - absolute.

---


### setPosition

```
public void setPosition(double position,
                        double sd)
```

:   Set the position of the locus on the linkage group in
    estimate mode i.e with uncertainty.

    :   **Specified by:**: `setPosition` in interface `IBioLocus`
    :   **Parameters:**: `position` - the locus position - absolute.


---


|  |  |  |
| --- | --- | --- |
| **Overview** \| **Package** \| **Class** \| **Use** \| **Tree** \| **Deprecated** \| **Index** \| **Help** | |  |
| **PREV CLASS**   **NEXT CLASS** | **FRAMES**    **NO FRAMES**     **All Classes** |  |
| SUMMARY: NESTED \| FIELD \| CONSTR \| METHOD | DETAIL: FIELD \| CONSTR \| METHOD |  |


---
